# 

**Published:** 1892-03

**Authors:** 


### CONTENTS—MARCH, 1892.—VOL. VII, No. 9.

ORIGINAL ARTICLES—

On Some Modifications of Barnes' Caoutchouc Uterine Dilators. By Prof. Wm. Keiller, F. R. C. S. Ed. Univ. Tex. Med. College, Galveston........ 309

Discussion on Prof. Keiller's Paper................. 314

Perityphlitis: W. R. Blailock, M. D., McGregor, Tex. 315

Treatment of the Morphine Habit: Jas. Johnston, M. D. 321

CORRESPONDENCE—

Clinical Notes from the N. Y. Hospital: Eugene Clark, M. D., Lockhart....... ............ ...... 325

SOCIETY NOTES—

Texas Medical Association 24th Annual Announcement ..................................... 328

Titles of Papers, Program, Etc..................... 329

EDITORIAL—

The Latest "Cure" for Consumption.. .............. 333

The Tick as a Cause of Cattle Fever.......... ..... 335

Watching the Watchman.......... ................ 338

MEDICAL NEWS AND MISCELLANY .................. 340

Antikamnia (Opposed to Pain) .................... 344

BOOK NOTICES.................................... 347

PUBLISHER'S NOTES ............. .............. 348

